# Combined occupational exposure to carcinogenic metals/metalloids and risk of lung cancer

**DOI:** 10.3389/fonc.2026.1772676

**Published:** 2026-04-06

**Authors:** Paolo Boffetta, David Zaridze, Beata Świątkowska, Tamás Pándics, Jolanta Lissowska, Eleonóra Fabiánová, John K. Field, Dana Mates, Miriam Schejbalová, Lenka Foretova, Vladimir Janout, Chengfeng Yang, Monireh Sadat Seyyedsalehi

**Affiliations:** 1 Stony Brook Cancer Center, Stony Brook University, Stony Brook, NY, United States; 2 Department of Family, Population and Preventive Medicine, Renaissance School of Medicine, Stony Brook University, Stony Brook, NY, United States; 3 Department of Medical and Surgical Sciences, University of Bologna, Bologna, Italy; 4 Department of Cancer Epidemiology and Prevention, N.N. Blokhin National Research Centre of oncology, Moscow, Russia; 5 Department of Environmental Epidemiology, The Nofer Institute of Occupational Medicine, Lodz, Poland; 6 National Center for Public Health and Pharmacy, Budapest, Hungary; 7 Department of Public Health Sciences, Faculty of Health Sciences, Semmelweis University, Budapest, Hungary; 8 Epidemiology Unit, Department of Cancer Epidemiology and Prevention, M. Sklodowska-Curie National Research Institute of Oncology, Warsaw, Poland; 9 Regional Authority of Public Health, Banská Bystrica, Slovakia; 10 Roy Castle Lung Cancer Research Programme, Department of Molecular and Clinical Cancer Medicine, University of Liverpool, Liverpool, United Kingdom; 11 National Institute of Public Health, Bucharest, Romania; 12 First Faculty of Medicine, Charles University, Prague, Czechia; 13 Masaryk Memorial Cancer Institute, Brno, Czechia; 14 Faculty of Health Sciences, Palacky University, Olomouc, Czechia; 15 Department of Pathology, Renaissance School of Medicine, Stony Brook University, Stony Brook, NY, United States

**Keywords:** arsenic, cadmium, chromium (VI), co-exposure, interaction, lung cancer, nickel

## Abstract

**Introduction:**

Exposure to arsenic, cadmium, chromium (VI) and nickel increases the risk of lung cancer; whereas humans are exposed to mixtures, epidemiology studies refer to individual metals/metalloids.

**Methods:**

We analysed the data of a case-control study of lung cancer conducted in seven European countries and comprising 2861 cases and 2936 controls, with detailed assessment of occupational exposure to arsenic, cadmium, chromium (VI) and nickel, to estimate the odds ratio (OR) of lung cancer for combined exposure to these metals/metalloids, after adjustment for potential confounders.

**Results:**

Odds ratios for combined exposure to arsenic and cadmium and to arsenic and chromium (VI) were higher than those for individual metals (in order of 1.2–1.4 for single metals and above 2.0 for combined exposure), although formal tests of interaction on additive and multiplicative scales were imprecise and compatible with no interaction. Estimates for combined exposure to chromium (VI) and nickel were lower than expected under additive or multiplicative models, but confidence intervals for the interaction metrics included the null. Results for co-exposure to three or four metals/metalloids were based on small numbers of exposed subjects.

**Discussion:**

Findings provide limited evidence of statistical interactions between occupational exposure to these metals in relation to lung cancer risk.

## Introduction

Exposure to several metals/metalloids including arsenic, cadmium, chromium (VI), and nickel has been associated with lung cancer in humans [1]. The strongest epidemiologic evidence arises from occupational settings with historically high exposure, such as metal smelting, electroplating, stainless-steel production, and alloy manufacturing [1]. Nonetheless, exposure to carcinogenic metals/metalloids also occurs in a broader range of industries including construction, welding, metalworking, and chemical manufacturing, as well as from environmental contamination, e.g., because of legacy industrial sites [2].

Most epidemiologic studies have examined individual metals/metalloids, despite the fact that workers are often co-exposed to multiple metals/metalloids simultaneously. This complicates the etiologic assessment because exposures are correlated and may interact biologically or through shared pathways of toxicity. Evaluating interactions in human studies remains challenging due to heterogeneous exposure circumstances, limited exposure detail, and insufficient statistical power to detect additive or multiplicative interaction [3].

Experimental studies provide mechanistic evidence that toxic metals/metalloids can interact. Co-exposure of lung epithelial cells to arsenic and chromium leads to synergistic increases in oxidative stress, DNA damage, and transformation, exceeding effects of either metal alone [4]. Chromium and nickel similarly show joint genotoxicity and chromatin alterations [5], while arsenic-transformed cells exhibit modified responses to nickel, indicating biological interaction [6]. Additional mixture studies involving arsenic–chromium–copper or chromium–nickel–cobalt systems demonstrate additive or supra-additive DNA damage and impaired repair [7; Jomova et al, 2024]. Together, these findings support the plausibility of synergistic or antagonistic metal–metal interactions relevant to lung carcinogenesis. Despite these insights, epidemiologic evidence directly evaluating co-exposure to multiple metals/metalloids is limited. An analysis based on the SYNERGY project evaluated joint effects for some occupational lung carcinogens, but did not distinguish the contribution of individual metals/metalloids [8]. Consequently, potential interactions among these four carcinogenic metals/metalloids remain poorly characterised in human populations.

To address this gap, we used data from a multicentre case–control study conducted in Central and Eastern Europe and the United Kingdom (UK), which collected detailed lifetime occupational histories and relied on expert-based assessment of exposure to 70 agents [3]. Results for exposure to arsenic, cadmium, chromium, and nickel were reported, without a systematic analysis of the effect of their combined exposures [9]. Given the historically higher prevalence of metal exposure in this region [10], these data provide an opportunity to evaluate whether co-exposure to arsenic, cadmium, chromium (VI), and nickel produces combined and interactive effects on lung cancer risk. These were the carcinogenic metals with the highest prevalence of exposure in the original multicentre study.

## Methods

The case-control study was conducted during 1998–2001 in 17 centres from 7 countries (Czech Republic, Hungary, Poland, Romania, Russia, Slovakia, and UK). Incident, histologically verified cases of lung cancer, including bronchi and trachea, were included, and controls were frequency-matched based on sex and age (± 3 years). Hospital-based controls were enrolled in all centres, except in Liverpool and Warsaw where population-based controls were selected. Hospital controls were selected from in-patients admitted for a pre-specified list of diseases which excluded other cancers and tobacco related diseases. Face-to-face interviews were conducted by trained interviewers using a questionnaire aiming on lifestyle factors and on occupations held for more than one year. A general questionnaire was administered for each job including questions on tasks performed, machines used, the working environment, and on the time spent on each task. A total of 18 specialised questionnaires were available to obtain more detailed information from subjects who had worked in one of the following occupations or industries: steel industry, coal gasification, foundry, glass industry, mechanic, wood worker, painter, welder, chemical industry, tannery, toolmaker and machine tool operator, miner or quarryman, insulation worker, printing, meat workers, farmer, rubber industry, asbestos compounds production. The questionnaires are included in **Supplementary Appendix A1**. The total interview typically lasted 45–60 minutes.

Industrial hygienists with expertise in the industries located in the study areas were involved in exposure assessment, which was based on a common protocol [3] derived from a previous study from Montréal, Canada [11]. This work consisted of evaluating for each job the exposure to the 70 agents, including metals/metalloids, based on the general questionnaire, the specialised questionnaires, if administered, and their own experience in the field. Indices to asses for each exposure included the expert’s confidence in the presence of the exposure (categorised as possible, probable or certain), the frequency of exposure defined as the percentage of working time exposed (categorised as 1-5%, 5-30%, >30%), and the intensity of exposure (categorised as low, medium and high on the basis of agent-specific cut-points). To obtain standardisation in the application of the intensity index, benchmarks were set consisting of jobs that would likely fall in either the low, medium or high intensity group. Wherever possible, the cut-points between low, medium and high intensity for each exposure were defined quantitatively based on measurements of the agent in the different jobs. Standardisation of the application of the exposure assessment methodology was endorsed through yearly workshops and coding exercises.

Unconditional logistic regression modelling was used to study the relation between pair-wise combinations of ever-exposure to arsenic, cadmium, chromium and nickel, and risk of lung cancer to calculate odds ratios (OR) and 95% confidence intervals (CI). All models were adjusted for age (three categories), centre, sex, and cumulative cigarette smoking (six categories of pack-years). Subjects were considered ever exposed if at least one job was assessed by the industrial hygienists as having possible, probable or certain exposure. In each pair-wise analysis, subjects unexposed to the two metals/metalloids comprised the reference category. Interactions between exposure to individual toxic metals/metalloids were evaluated on both multiplicative and additive scales using the interaction odds ratio (IR) and the relative excess risk due to interaction (RERI), respectively; 95% confidence intervals were calculated for both indicators [12]. Confidence intervals for interaction metrics including the null values (IR = 1 or RERI = 0, respectively) were interpreted as indicating no clear statistical evidence of interaction.

In addition, exploratory analyses were conducted on combined exposure to three and four metals/metalloids, using subjects unexposed to all four metals/metalloids as reference category: the regression models were similar to those described above for the pairwise comparisons. These analyses were considered exploratory because of the low expected number of subjects in the different categories of combinations of exposure. The number of subjects in the combined exposure categories was sufficient for analyses based on ever-exposure to each agent, but too low for analyses based on semi-quantitative indices of exposure to metals/metalloids.

Different approaches were also explored for the categorisation of cigarette smoking, which however did not alter the results on metal exposure. Given the relatively low number of subjects exposed to the different combinations of toxic metals/metalloids, no stratified analyses were performed, e.g., by gender, age of diagnosis, histological type or stage of lung cancer or country. We performed two sensitivity analyses, one in which the reference categories comprised only subjects not exposed to any of the four agents included in the analysis, and one in which exposure to asbestos, crystalline silica, wood dust and polycyclic aromatic hydrocarbons was adjusted for.

All analyses were performed using STATA package, Version 17.0. The study was approved by the relevant Ethics Committee.

## Results

**Table 1** provides details on the study population. Overall, 2861 cases and 2936 controls were included in the analysis, with a majority of men (77.1% of cases, 73.7% of controls). The median age at enrolment was 61 for both cases (interquartile range: 53-68) and controls (54-68). As expected, cigarette smoking, especially heavy smoking, was more prevalent among cases: the OR for the categories of pack-years shown in **Table 1**, compared to never smokers, were 2.70, 6.99, 10.57, 11.86, and 17.70, respectively.

**Table 1:** Selected characteristics of the study population.

Characteristic	Category	Cases		Controls	
		N	%	N	%
Total		2861	100.0	2936	100.0
Sex	Male	2205	77.1	2164	73.7
	Female	656	22.9	772	26.3
Country	Czech Republic	304	10.6	453	15.4
	Hungary	402	14.1	315	10.7
	Poland	800	28.0	841	28.6
	Romania	181	6.3	228	7.8
	Russia	600	21.0	600	19.3
	Slovakia	346	12.1	285	9.7
	United Kingdom	228	8.0	234	8.0
Age (years)	25-54	750	26.2	828	28.2
	55-64	1042	36.4	973	33.1
	65+	1069	37.4	1135	38.7
Cumulative cigarette smoking (pack-years)	0 (Never smoker)	274	9.6	1041	35.5
	0.1-19.9	422	14.7	754	25.7
	20.0-29.9	526	18.4	394	13.4
	30.0-39.9	646	22.6	341	11.6
	40.0-49.9	480	16.8	230	7.8
	50.0+	513	17.9	176	6.0

The prevalence of ever-occupational exposure among controls was 2.3% for arsenic, 3.5% for cadmium, 8.7% for chromium (VI) and 4.9% nickel. lifetime occupational exposure prevalence for metals/metalloids appeared to be high in this population. Within the group of male controls, 11.4% was ever exposed to chromium, 6.4% to nickel, 3.9% to cadmium, 1.7% to arsenic and 14.0% to any of these metals/metalloids. Exposure to chromium, cadmium and nickel occurred mainly in the construction and metal manufacturing industries, while exposure to arsenic mainly occurred in agriculture and the chemical industry.

The correlation coefficients among controls between the toxic metals/metalloids under study are reported in **Table 2**. The highest correlation was found for cadmium and chromium (VI) and for chromium (VI) and nickel. In particular, 68% of controls exposed to cadmium and 73% of controls exposed to nickel were also exposed to chromium (VI), respectively.

**Table 2:** Pearson correlation coefficients between ever-exposure to toxic metals under study (in parentheses, percentage of those exposed to the agent in the column, who are also exposed to the agent in the row) – Analysis restricted to controls.

Metal exposure	Arsenic	Cadmium	Chromium (VI)	Nickel
Arsenic	–	0.25 (22%)	0.16 (10%)	0.14 (12%)
Cadmium	0.25 (34%)	–	0.41 (28%)	0.26 (25%)
Chromium (VI)	0.16 (39%)	0.41 (68%)	–	0.52 (73%)
Nickel	0.14 (25%)	0.26 (35%)	0.52 (23%)	–

The results of the analysis of co-exposure to pairs of metals/metalloids are reported in **Table 3**. For both combined exposure to arsenic and cadmium [IR = 1.19 (95%CI=0.55-2.58), RERI = 0.49 (95%CI=-0.91-1.88)], and to arsenic and chromium (VI) [IR = 1.29 (95%CI=0.63-2.64), RERI = 0.56 (95%CI=-0.04-0.30)], the OR were greater than the products of the OR of individual metals/metalloids (IR > 1.0), compatible with interaction according to the multiplicative model; whereas the OR for exposure to a single metal were in the order 1.2-1.4, the OR for combined exposure exceeded 2.0. However, the confidence intervals for both interaction metrics included the null and did not provide clear evidence of interaction.

**Table 3:** Odds ratio of lung cancer for exposure to pairwise combinations of toxic metals/metalloids.

Combination of toxic metals/metalloids	Cases		Controls		OR	95% CI	RERI		IR	
	N	%	N	%			Value	95% CI	Value	95%CI
Arsenic and cadmium										
Only arsenic	51	1.8	44	1.5	1.31	0.83-2.06				
Only cadmium	104	3.6	81	2.8	1.41	1.01-1.97				
Both	49	1.7	23	0.8	2.20	1.27-3.81	0.49	-0.91,1.88	1.19	0.55-2.58
Arsenic and chromium (VI)										
Only arsenic	47	1.6	41	1.4	1.32	0.82-2.11				
Only chromium (VI)	281	9.8	228	7.8	1.25	1.02-1.54				
Both	53	1.9	26	0.9	2.13	1.26-3.59	0.56	-0.04,0.30	1.29	0.63-2.64
Arsenic and nickel										
Only arsenic	70	2.5	50	1.7	1.54	1.02-2.34				
Only nickel	156	5.5	127	4.3	1.16	0.89-1.52				
Both	30	1.1	17	0.6	1.79	0.93-3.45	0.08	-1.26,1.43	1.00	0.45-2.23
Cadmium and chromium (VI)										
Only cadmium	48	1.7	33	1.1	1.55	0.94-2.55				
Only chromium (VI)	229	8.0	183	6.2	1.24	0.99-1.55				
Both	105	3.7	71	2.4	1.64	1.16-2.31	-0.15	-1.12,0.83	0.86	0.45-1.61
Cadmium and nickel										
Only cadmium	90	3.1	68	2.3	1.55	1.08-2.23				
Only nickel	123	4.3	108	3.7	1.10	0.82-1.47				
Both	63	2.2	36	1.2	1.64	1.04-2.58	-0.01	-0.97,0.96	0.97	0.51-1.83
Chromium (VI) and nickel										
Only chromium (VI)	199	7.0	149	5.1	1.43	1.12-1.84				
Only nickel	51	1.8	39	1.3	1.37	0.86-2.17				
Both	135	4.7	105	3.6	1.21	0.91-1.62	-0.59	-1.28,0.21	0.62	0.34-1.18

Reference category, for each analysis, subjects unexposed to both metals/metalloids.

OR, odds ratio, adjusted for centre, sex, age, cumulative tobacco smoking.

CI, confidence interval.

RERI, relative excess risk due to interaction.

IR, interaction ratio.

For cadmium and chromium (VI) [IR = 0.86 (95%CI=0.45-0.61), RERI =-0.15 (95% CI=-1.12-0.83] and chromium (VI) and nickel [IR = 0.62 (95% CI = 0.34-1.18, RERI = -0.59 (95% CI=-1.28-0.21], the OR for combined exposure were lower than expected according to either the multiplicative or additive model, but none of the interaction metrics for these pairs were statistically significant.

Results of the sensitivity analysis in which the reference category comprised only subjects not exposed to any of the four metals included in this analysis yielded results very similar to those of the primary analysis: as an example, the OR for the combined exposure to arsenic and chromium (VI) were 1.33 (95% CI 0.83-2.15), 1.27 (95% CI 1.03-1.56) and 2.16 (95% CI 1.28-3.64) for exposure to arsenic, chromium (VI) and both agents, respectively.

The results of the exploratory analysis of exposure to three or four metals/metalloids are reported in **Supplementary Table 1**. As expected, the number of subjects included in some of the categories was too low to produce sufficiently precise results. The OR for exposure to all four metals/metalloids, compared to exposure to none, was 2.44 (95% CI 1.03-5.75), based on 20 cases and 9 controls.

The results of the sensitivity analysis in which each interaction term was adjusted for exposure to other carcinogens are reported in **Supplementary Table 2**. They are similar to the results in which terms for exposure to other carcinogens were not included in the regression models (**Table 3**).

## Discussion

This multi-centre study, including almost 3000 cases and 3000 controls from Central/Eastern Europe and the UK, is one of the largest studies of lung cancer with detailed information on occupational exposures, allowing a detailed analysis of co-exposure to more than one toxic metal/metalloid. The results showed higher odds ratios for combined exposure to arsenic and cadmium and to arsenic and chromium (VI) than for individual metals. However, formal measures of interaction on both the multiplicative and additive scales were imprecise and compatible with no interaction. Therefore, the findings do not provide clear statistical evidence of interaction but suggest patterns that may warrant further investigation. For chromium (VI) and nickel, the point estimates for combined exposure were lower than expected under additive or multiplicative models; however, the confidence intervals included the null, and therefore the data do not provide clear evidence of antagonistic interaction. For the other combinations, the results indicated a higher OR for those exposed to both metals/metalloids compared to those exposed to each of them individually but did not allow to distinguish between the additive and the multiplicative model.

Few epidemiologic investigations have directly quantified metal–metal interactions for lung cancer. The SYNERGY pooled analysis of 18,000 cases and 21,000 controls evaluated pairwise interactions between asbestos, crystalline silica, PAHs, chromium (VI), and nickel, but chromium (VI) and nickel were not separated and arsenic and cadmium were not included [8]. Thus, our findings provide novel evidence regarding arsenic-based interactions, which remain largely unexplored in human populations. Similarly, a study from Montréal included in the SYNERGY collaboration (13) has reported increased risks for individual metals/metalloids but limited interaction analysis; these analyses suggested potential multi-metal effects involving chromium (VI), nickel, and cadmium but were limited by small sample sizes, consistent with our observation that larger studies are needed to confirm interactions. The ATSDR Interaction Profile for arsenic, cadmium and chromium concluded that epidemiologic evidence for metal–metal interactions is sparse despite substantial mechanistic evidence [2], consistent with the knowledge gap addressed by the present study.

The potential mechanisms responsible for the increased lung cancer risk observed for arsenic and cadmium or for arsenic and chromium (VI) could include synergistic epigenetic dysregulation, as studies have shown that arsenic, cadmium, or chromium (VI) exposures cause distinct epigenetic alterations [14–16]. In addition, co-exposure may therefore produce more extensive or stronger dysregulation, enhancing malignant transformation. In addition, arsenic and cadmium or arsenic and chromium (VI) may act synergistically to cause epitranscriptomic dysregulations, as these metals/metalloids alter RNA modifications and regulatory pathways [17, 18]. Combined exposures may accelerate dysregulation of mRNA stability, translation, and noncoding RNA networks, promoting lung cancer development. Furthermore, arsenic may amplify the genotoxic effects of cadmium and chromium (VI) by inhibiting DNA damage repair, increasing the accumulation of unrepaired lesions, which is consistent with experimental observations of enhanced DNA damage and chromosomal instability in cells co-exposed to arsenic and chromium (VI) or cadmium. Finally, co-exposure may synergize in activating oncogenic signalling pathways, including PI3K/AKT, MAPK/ERK, and oxidative stress responses, which could facilitate malignant transformation and lung cancer progression.

Findings from *in vitro* studies support these mechanistic hypotheses: co-exposure to arsenic and chromium (VI) in human bronchial epithelial cells produces marked synergy in oxidative stress, PI3K/AKT activation, DNA damage, and cellular transformation, effects not observed at comparable single-metal doses [4]. Another *in vitro* study showed that combined exposure to arsenite and cadmium rapidly activated MAPK/ERK signalling via estrogen receptor (ER) and G-protein-coupled ER (GPER) in human lung adenocarcinoma cells at environmentally relevant concentrations, effects not seen with single-metal exposure alone [19]. Additional mixture studies involving chromium, nickel, and arsenic report greater-than-additive DNA damage and impaired repair [4, 7], and multi-metal reviews describe persistent oxidative stress and genotoxicity beyond single-metal expectations [20], supporting the biological plausibility of the interactions observed in the present study. The sub-additive interaction between chromium (VI) and nickel may reflect biological antagonism, potentially due to competition for cellular uptake, altered metal speciation, or adaptive stress responses, such as arsenic-induced cross-tolerance to nickel cytotoxicity [6]. Because chromium (VI) and nickel partly target different molecular pathways, combined exposure may not amplify and may even interfere with each other’s effects. Experimental work on chromium–nickel mixtures has reported both additive and antagonistic outcomes, consistent with the epidemiologic findings [21, 22].

This study has several potential limitations. Although the overall sample size was large, the number of subjects exposed to specific multi-metal/metalloid combinations was limited, reducing precision for analyses of three- or four-metal co-exposures, and preventing interaction analyses based on semi-quantitative indices of exposure. In addition, albeit the OR for exposure to single metal/metalloid were increased, as expected given the known carcinogenicity of these agents, in some instances they did not reach the canonical level of statistical significance, The use of hospital-based controls may introduce selection bias; however, previous analyses from this population reported risk estimates for smoking, indoor air pollution, and various occupational exposures [9, 23–25] replicated established associations, suggesting limited opportunity for bias. Residual confounding cannot be entirely excluded, although additional adjustment for education and other occupational exposures did not materially change results. Some exposure misclassification is likely, but because assessment was performed blind to disease status, it is expected to be non-differential and would likely attenuate rather than inflate associations or create spurious interactions. This phenomenon would affect both positive (synergistic) and negative (antagonistic) interaction analyses. Routes of exposure were not considered separately; however, most of exposures were likely to be via inhalation. Smoking was addressed using detailed lifetime pack-year categories and with alterative categorizations; while minor residual confounding is possible, it is unlikely to explain the observed interaction patterns.

In conclusion, this study helped inform joint effects of occupational exposure to arsenic, cadmium, chromium (IV), and nickel in relation to lung cancer risk. Although the odds ratios for some combined exposures were higher than those for individual metals, formal tests of interaction on both the additive and multiplicative scales were not statistically significant. Therefore, findings provide limited evidence of statistical interactions between the metals that were evaluated. These findings emphasise the importance of evaluating realistic co-exposure scenarios in occupational settings, while also underscoring the need for larger studies with sufficient statistical power to more definitively assess potential interactions among carcinogenic metals.

## Data Availability

The raw data supporting the conclusions of this article will be made available by the authors, without undue reservation.

## Supplementary material

Supplementary Table 1 Odds ratio of lung cancer for exposure to combinations of toxic metals/metalloids Reference category: subjects unexposed to all four metals/metalloids. As, arsenic; Cd, cadmium; Cr(VI), chromium (VI), NI, nickel. OR, odds ratio, adjusted for centre, sex, age, cumulative tobacco smoking (not reported for categories with < 5 exposed cases and controls). CI, confidence interval

Supplementary Table 2 Odds ratio of lung cancer for exposure to pairwise combinations of toxic metals/metalloids, with adjustment for exposure to other carcinogens Reference category: for each analysis, subjects unexposed to both metals/metalloids OR, odds ratio, adjusted for centre, sex, age, cumulative tobacco smoking, and exposure to asbestos, silica, wood dust and polycyclic aromatic hydrocarbons CI, confidence interval

## References

[1] International Agency for Research on Cancer . Arsenic, metals, fibres, and dusts. In: IARC monogra[hs on the evaluation of carcinogenic risks to humans, vol. 100. IARC, Lyon (2012). PMC4781271 23189751

[2] Agency for Toxic Substances and Disease Registry (ATSDR) . Interaction profile for arsenic, cadmium, chromium, and lead. Washington, DC, U.S: Department of Health and Human Services, Public Health Service (2004).

[3] ‘t Mannetje A Fevotte J Fletcher T Brennan P Legoza J Szeremi M . Assessing exposure misclassification by expert assessment in multicenter occupational studies. Epidemiology. (2003) 14:585–92. doi: 10.1097/01.ede.0000072108.66723.0f, PMID: 14501274

[4] Ohgami N Yamanoshita O Thang ND Yajima I Nakano C Wenting W . Carcinogenic risk of chromium, copper and arsenic in CCA-treated wood. Environ pollut. (2015) 206:456–60. doi: 10.1016/j.envpol.2015.07.041, PMID: 26275730

[5] Behrens T Ge C Vermeulen R Kendzia B Olsson A Schüz J . Occupational exposure to nickel and hexavalent chromium and the risk of lung cancer in a pooled analysis of case-control studies (SYNERGY). Int J Cancer. (2023) 152:645–60. doi: 10.1002/ijc.34272, PMID: 36054442

[6] Qu W Kasprzak KS Kadiiska M Liu J Chen H Maciag A . Mechanisms of arsenic-induced cross-tolerance to nickel cytotoxicity, genotoxicity, and apoptosis in rat liver epithelial cells. Toxicol Sci. (2001) 63:189–95. doi: 10.1093/toxsci/63.2.189, PMID: 11568362

[7] Papageorgiou I Yin Z Ladon D Baird D Lewis AC Sood A . Genotoxic effects of particles of surgical cobalt chrome alloy on human cells of different age in *vitro.* Mutat. Res. (2007) 619:45–58. doi: 10.1016/j.mrfmmm.2007.01.008, PMID: 17376492

[8] Olsson A Bouaoun L Schüz J Vermeulen R Behrens T Ge C . Lung cancer risks associated with occupational exposure to pairs of five lung carcinogens: Results from a pooled analysis of case-control studies (SYNERGY). Environ Health Perspect. (2024) 132:17005. doi: 10.1289/EHP13380, PMID: 38236172 PMC10795675

[9] ‘t Mannetje A Bencko V Brennan P Zaridze D Szeszenia-Dabrowska N Rudnai P . Occupational exposure to metal compounds and lung cancer: Results from a multi-center case-control study in Central/Eastern Europe and UK. Cancer Causes. Ctrl. (2011) 22:1669–80. doi: 10.1007/s10552-011-9843-3, PMID: 21960145

[10] Fabiánová E Szeszenia-Dabrowska N Kjaerheim K Boffetta P . Occupational cancer in central European countries. Environ Health Perspect. (1999) 107:279–82. doi: 10.1289/ehp.107-1566282, PMID: 10350511 PMC1566282

[11] Siemiatycki J Wacholder S Richardson L Dewar R Gérin M . Discovering carcinogens in the occupational environment: Methods of data collection and analysis of a large case-referent monitoring system. Scand J Work. Environ Health. (1987) 13:486–92. doi: 10.5271/sjweh.2009, PMID: 3433050

[12] VanderWeele TJ Knol MJ . A tutorial on interaction. Epidemiol. Meth. (2014) 3:33–72. doi: 10.1515/em-2013-0005, PMID: 41717541

[13] Beveridge R Pintos J Parent ME Asselin J Siemiatycki J . Lung cancer risk associated with occupational exposure to nickel, chromium VI, and cadmium in two population-based case-control studies in Montreal. Am J Ind Med. (2010) 53:476–85. doi: 10.1002/ajim.20801, PMID: 20187007

[14] Wang Z Wang PS Yang C . Dysregulation of long non-coding RNAs—the novel lnc in metal toxicity and carcinogenesis. Curr Environ Health Rep. (2024) 12:3. doi: 10.1007/s40572-024-00468-1, PMID: 39715843 PMC11755759

[15] Zhu Y Costa M . Metals and molecular carcinogenesis. Carcinogenesis. (2020) 41:1161–72. doi: 10.1093/carcin/bgaa076, PMID: 32674145 PMC7513952

[16] Wang PS Wang Z Yang C . Dysregulations of long non-coding RNAs—the emerging “lnc” in environmental carcinogenesis. Semin Cancer Biol. (2021) 76:163–72. doi: 10.1016/j.semcancer.2021.03.029, PMID: 33823237 PMC8487435

[17] Yang C Wang Z . The epitranscriptomic mechanism of metal toxicity and carcinogenesis. Int J Mol Sci. (2022) 23:11830. doi: 10.3390/ijms231911830, PMID: 36233132 PMC9569618

[18] Wang Z Yang C . Epigenetic and epitranscriptomic mechanisms of chromium carcinogenesis. Adv Pharmacol. (2023) 96:241–65. doi: 10.1016/bs.apha.2022.07.002, PMID: 36858774 PMC10565670

[19] Huff MO Todd SL Smith AL Elpers JT Smith AP Murphy RD . Arsenite and cadmium activate MAPK/ERK via membrane estrogen receptors and G-protein coupled estrogen receptor signaling in human lung adenocarcinoma cells. Toxicol Sci. (2016) 152:62–71. doi: 10.1093/toxsci/kfw064, PMID: 27071941

[20] Jomova K Alomar SY Nepovimova E Kuca K Valko M . Heavy metals: toxicity and human health effects. Arch Toxicol. (2025) 99:153–209. doi: 10.1007/s00204-024-03903-2, PMID: 39567405 PMC11742009

[21] Lou J Jin L Wu N Tan Y Song Y Gao M . DNA damage and oxidative stress in human B lymphoblastoid cells after combined exposure to hexavalent chromium and nickel compounds. Food Chem Toxicol. (2013) 55:533–40. doi: 10.1016/j.fct.2013.01.053, PMID: 23410589

[22] Permenter MG Lewis JA Jackson DA . Exposure to nickel, chromium, or cadmium causes distinct changes in the gene expression patterns of a rat liver-derived cell line. PloS One. (2011) 6:e27730. doi: 10.1371/journal.pone.0027730, PMID: 22110744 PMC3218028

[23] Brennan P Crispo A Zaridze D Szeszenia-Dabrowska N Rudnai P Lissowska J . High cumulative risk of lung cancer death among smokers and nonsmokers in Central and Eastern Europe. Am J Epidemiol. (2006) 164:1233–41. doi: 10.1093/aje/kwj340, PMID: 17032696

[24] Lissowska J Bardin-Mikolajczak A Fletcher T Zaridze D Szeszenia-Dabrowska N Rudnai P . Lung cancer and indoor pollution from heating and cooking with solid fuels: the IARC international multicentre case-control study in Eastern/Central Europe and the United Kingdom. Am J Epidemiol. (2005) 162:326–33. doi: 10.1093/aje/kwi204, PMID: 16014775

[25] ‘t Mannetje A Brennan P Zaridze D Szeszenia-Dabrowska N Rudnai P Lissowska J . Welding and lung cancer in Central and Eastern Europe and the United Kingdom. Am J Epidemiol. (2012) 175:706–14. doi: 10.1093/aje/kwr358, PMID: 22343633

