# Supplementary material for: Combined occupational exposure to carcinogenic metals/metalloids and risk of lung cancer
Source: Front Oncol. 2026 Apr 6;16:1772676. doi: 10.3389/fonc.2026.1772676 (PMC13093972; doi:10.3389/fonc.2026.1772676)
Supplement: Supplementary Table 1 — Odds ratio of lung cancer for exposure to combinations of toxic metals/metalloids Reference category: subjects unexposed to all four metals/metalloids. As, arsenic; Cd, cadmium; Cr(VI), chromium (VI), NI, nickel. OR, odds ratio, adjusted for centre, sex, age, cumulative tobacco smoking (not reported for categories with < 5 exposed cases and controls). CI, confidence interval [file Table1.docx]

**Supplementary Table 1.** Odds ratio of lung cancer for exposure to combinations of toxic metals/metalloids

| Combination of metals/metalloids | Cases | | Controls | | OR | 95% CI |
| --- | --- | --- | --- | --- | --- | --- |
|  | N | % | N | % |  |  |
| Only As | 38 | 1.3 | 32 | 1.1 | 1.40 | 0.82-2.37 |
| Only Cd | 26 | 0.9 | 19 | 0.7 | 1.50 | 0.77-2.95 |
| Only Cr(VI) | 131 | 4.6 | 96 | 3.3 | 1.39 | 1.03-1.87 |
| Only Ni | 30 | 1.1 | 22 | 0.7 | 1.50 | 0.82-2.74 |
| As, Cd | 3 | 0.1 | 2 | 0.1 | - | - |
| As, Cr(VI) | 7 | 0.2 | 6 | 0.2 | 1.57 | 0.47-5.21 |
| As, Ni | 2 | 0.1 | 5 | 0.2 | - | - |
| Cd, Cr(VI) | 39 | 1.4 | 37 | 1.3 | 1.44 | 0.86-2.41 |
| Cd, Ni | 15 | 0.5 | 10 | 0.3 | 1.49 | 0.63-3.53 |
| Cr(VI), Ni | 87 | 3.0 | 80 | 2.7 | 1.04 | 0.74-1.47 |
| As, Cd, Cr(VI) | 22 | 0.8 | 10 | 0.3 | 2.19 | 0.98-4.91 |
| As, Cd, Ni | 4 | 0.1 | 2 | 0.1 | - | - |
| As, Cr(VI), Ni | 4 | 0.1 | 1 | 0.03 | - | - |
| Cd, Cr(VI), Ni | 24 | 0.8 | 15 | 0.5 | 1.37 | 0.67-2.77 |
| As, Cd, Cr(VI), Ni | 20 | 0.7 | 9 | 0.3 | 2.44 | 1.03-5.75 |

Reference category: subjects unexposed to all four metals/metalloids

As, arsenic; Cd, cadmium; Cr(VI), chromium (VI), NI, nickel

OR, odds ratio, adjusted for center, sex, age, cumulative tobacco smoking (not reported for categories with < 5 exposed cases and controls)

CI, confidence interval
