# Supplementary material for: Combined occupational exposure to carcinogenic metals/metalloids and risk of lung cancer
Source: Front Oncol. 2026 Apr 6;16:1772676. doi: 10.3389/fonc.2026.1772676 (PMC13093972; doi:10.3389/fonc.2026.1772676)
Supplement: Supplementary Table 2 — Odds ratio of lung cancer for exposure to pairwise combinations of toxic metals/metalloids, with adjustment for exposure to other carcinogens Reference category: for each analysis, subjects unexposed to both metals/metalloids OR, odds ratio, adjusted for centre, sex, age, cumulative tobacco smoking, and exposure to asbestos, silica, wood dust and polycyclic aromatic hydrocarbons CI, confidence interval [file Table2.docx]

**Supplementary Table 2**. Odds ratio of lung cancer for exposure to pairwise combinations of toxic metals/metalloids, with adjustment for exposure to other carcinogens

| Combination of toxic metals/metalloids | OR | 95% CI |
| --- | --- | --- |
| Arsenic and cadmium |  |  |
| Only arsenic | 1.24 | 0.78-1.96 |
| Only cadmium | 1.36 | 0.97-1.90 |
| Both | 2.08 | 1.20-3.61 |
|  |  |  |
| Arsenic and chromium (VI) |  |  |
| Only arsenic | 1.26 | 0.92-1.36 |
| Only chromium (VI) | 1.21 | 0.98-1.49 |
| Both | 1.98 | 1.17-3.34 |
|  |  |  |
| Arsenic and nickel |  |  |
| Only arsenic | 1.65 | 0.96-2.20 |
| Only nickel | 1.17 | 0.89-1.52 |
| Both | 1.74 | 0.90-3.36 |
|  |  |  |
| Cadmium and chromium (VI) |  |  |
| Only cadmium | 1.52 | 0.94-2.51 |
| Only chromium (VI) | 1.20 | 0.95-1.50 |
| Both | 1.55 | 1.10-2.20 |
|  |  |  |
| Cadmium and nickel |  |  |
| Only cadmium | 1.46 | 1.02-2.11 |
| Only nickel | 1.11 | 0.82-1.48 |
| Both | 1.61 | 1.02-2.54 |
|  |  |  |
| Chromium (VI) and nickel |  |  |
| Only chromium (VI) | 1.34 | 1.05-1.72 |
| Only nickel | 1.35 | 0.85-2.15 |
| Both | 1.21 | 0.90-1.62 |

Reference category: for each analysis, subjects unexposed to both metals/metalloids

OR, odds ratio, adjusted for center, sex, age, cumulative tobacco smoking, and exposure to asbestos, silica, wood dust and polycyclic aromatic hydrocarbons

CI, confidence interval
