# Supplementary material for: Combined occupational exposure to carcinogenic metals/metalloids and risk of lung cancer
Source: Front Oncol. 2026 Apr 6;16:1772676. doi: 10.3389/fonc.2026.1772676 (PMC13093972; doi:10.3389/fonc.2026.1772676)
Supplement: Supplementary file 3 [file SupplementaryFile1.docx]

**Appendix 1.** Occupational questionnaires

*Note : The format of the original questionnaire was changed in order to save space*

| **JOB HISTORY**  **(JH)** | **Identification number:** |
| --- | --- |
| Please list below all the jobs you have held starting with the first job you had after leaving general school. Include all major job changes within the same company as separate jobs, (e.g. permanent moves to different sectors, any promotions). Ignore jobs you held for less than 1 year but include all periods without any job (house keeping, apprenticeship, military service, working on family farm/ business) if they were lasting at least 1 year. | |
| Job number 1 from year: to year : | occupation/job title:  company/employer name: city:  activity/production:  If there is a period of more than 1 year without occupation before job number 2, please note the reason: |
| Job number 2 from year: to year : | occupation/job title:  company/employer name: city:  activity/production:  If there is a period of more than 1 year without occupation before job number 3, please note the reason: |
| Job number 3 from year: to year : | occupation/job title:  company/employer name: city:  activity/production:  If there is a period of more than 1 year without occupation before job number 4, please note the reason: |
| **Job number 4**  from year: to year : | occupation/job title:  company/employer name: city:  activity/production:  If there is a period of more than 1 year without occupation before job number 5, please note the reason: |
| **Job number 5**  from year: to year : | occupation/job title:  company/employer name: city:  activity/production:  If there is a period of more than 1 year without occupation before job number 6, please note the reason: |
| Job number 6 from year: to year : | occupation/job title:  company/employer name: city:  activity/production:  If there is a period of more than 1 year without occupation before job number 7, please note the reason: |
| Job number 7 from year: to year : | occupation/job title:  company/employer name: city:  activity/production:  If there is a period of more than 1 year without occupation before job number 8, please note the reason: |
| Job number 8 from year: to year : | occupation/job title:  company/employer name: city:  activity/production:  If you have additional employment periods, please fill them in on an additional page. |
| During any job you have held, did you have to stop working for more than 1 year because of illness?  if yes, for which job? job number : and when? from year : to year : | |
|  | **GO TO EJ (EXTRA JOBS)** |

| *EXTRA JOBS* *(EJ)* | Identification number: |
| --- | --- |
| *Note to Interviewers: After obtaining the full job history, please ask the patient whether he/she had any extra/unofficial jobs which involved any of the following five tasks for which they received payment. If possible, complete a GQ for these extra jobs (but not a SQ).* | |
| *Building/construction* yes/no | From year: to year: n hours a week: What did you do?: |
| *Painting* yes/no | From year: to year: n hours a week: What did you do?: |
| *Welding* yes/no | From year: to year: n hours a week: What did you do?: |
| *Wood working* yes/no | From year: to year: n hours a week: What did you do?: |
| *Agriculture* yes/no | From year: to year: n hours a week: Which ones?: |
| *Animal breeding* yes/no | From year: to year: n hours a week: Which animals?: |
| *Transport* yes/no | From year: to year: n hours a week: What did you do?:  transport of passengers (incl taxis),  or of freight (and which kind of freight) |
| *COMPLETE A GQ FOR EACH JOB IN THE JH (AND IN THE EJ IF POSSIBLE)* | |

| **GENERAL QUESTIONNAIRE (GQ)** | Identification number:Job number: from year: to year: |
| --- | --- |
|  |  |
| **Q1**: was it a full time job?…………. | yes/noif part-time, how many hours a week? |
| **Q2**: Was it a seasonal job? ……… | yes/no  if seasonal, how many months a year?  and for how many years ? |
| **Q3**: Can you describe in more detail the activities or products made by your company or employer?………. | describe: |
| **Q4**: In which sector (department) of activity or production were you personally involved?………………. | describe: |
| **Q5**: Can you describe the place (room) where you usually worked? | indoor / outdoor / underground / at home / in an office /  in a laboratory / in a warehouse / in a plant / in a vehicle /  if other, please specify:  *In case of job outdoors only, please go straight to Q7* |
| **Q6**: How many people worked in this same place? …………………… | describe: |
| **Q7**: Please, describe your specific tasks (what you did, and how you did it). …………………………………  *(If you performed different tasks, please start with the main one, the most time consuming)* | describe: |
| **Q8**: How much time did you spend on your main task ?…………….…..  (% of the day or of the week or of the month, or hours/day, hours/week, days/week: please specify) | describe: |
| **Q9**: Which machine or equipment did you use ? | describe: |
| If you used machines did you clean or maintain them ?…………………. | yes/no  if yes, how many hours per week:  if yes, describe how you did it: |
| **Q10**: What kind of different jobs were done by others working nearby to you ?……………………… | describe: |
| *Note for interviewers : in case of clerical work without any possibility of exposure, please go straight to the next job description* |  |
| **Q11**: Were you exposed to dust ?… | Y/N/DK Hours/week Source of exposure  •sand  •cement  •concrete  •metal  •wood  •coal  •soot  if other, please specify: |
| **Q12**: Were you exposed to fumes? | Y/N/DK Hours/week Source of exposure  •diesel engine  •gasoline engine  •heating equipment  •coal combustion  •coke combustion  •fuel combustion  •wood combustion  if other, please specify: |
| **Q13**: Were you exposed to oils?.… | Y/N/DK Hours/week Source of exposure  •lubricating oil  •cutting fluids  •mineral oils  if other, please specify: |
| **Q14**: Were you exposed to plastics or synthetic rubbers?.…………..….. | Y/N/DK Hours/week Source of exposure  •PVC (polyvinyl chloride)  •Polyacrylonitrile  •ABS rubber(Acrylonitrile-butadiene-styrene)  •SAN rubber (stryene-acrylonitrile)  •reinforced plastic polyesters  if other, please specify: |
| **Q15**: Which of the following did you handle or were you exposed to?.… | Y/N/DK description/name Hours/week Source of exposure  •pesticides  •wood preservatives  •caulking, sealants  •acids  •adhesives  •cleaning agents  •disinfecting agents  if yes, was it NaCIO *(note for translation: use usual name)*  if other, please specify: |
| **Q16**: What other materials or chemicals than those described before, did you use or were you exposed to ? ………………………..  *For each one, can you specify their use or function (e.g. raw material, colouring agent,...) and how much time you used it (or were exposed to it)* | description/name hours/week source exposure  •  •  • |
| **Q17**: Was there any significant change in your tasks or in products you used during this period?.……… | yes/no  if yes, describe what the change was and when: |
| **Q18**: Did you do any welding or gas cutting?………………………... | yes/no  if yes, hours a week:  if no, was gas welding or gas cutting done near you by others?  yes/no  if yes, hours a week :  was arc welding done near you by others ?  yes/no  if yes, hours a week:  *if yes, please go to Q25 and check to welding Specific Questionnaire that you will fill after ending this one, then go to Q19* |
| **Q19**: Did you do any painting ?….. | yes/no  if yes, hours a week:  if no, was painting done near you by others?  yes/no  if yes, hours a week:  *If yes, please go to Q25 and check to painting Specific Questionnaire that you will fill after ending this one, then go to Q20* |
| **Q20**: Did you install or remove or work with any kind of insulation or fibre panels?.……………………….. | yes/no  if yes, hours a week:  if no, do any people install or remove or work with insulation or fiber panels near you ?  yes/no  if yes, hours a week:  *if yes, please go to Q25 and check to insulation Specific Questionnaire that you will fill after ending this one, then go to Q21* |
| **Q21**: Were you exposed to ionizing radiation?……………………………. | yes/no  if yes, hours a week:  if yes, give the reason for exposure: |
| **Q22**: Were you exposed to radioactive material?………………. | yes/no  if yes, hours a week:  if yes, description/name of materials and source/reason of exposure: |
| **Q23**: Did you use any individual protective equipment ? If yes, for which tasks?.……………………….. | yes/no tasks  •simple dust mask  •air supplied mask  •glasses  •asbestos made equipment (gloves, screens)  •leather made equipment  if other, please specify: |
| **Q24**: Was there any local exhaust ventilation in the place where you usually worked? ……….……..…... | yes/no  if yes, for which tasks? |
| **Q25**: *Note to Interviewer : Go through the following list and tick the specialised questionnaires which need to be completed for the current job. Then proceed to complete the SQs before moving on to the next job.* | 1- steel industry  2- coke manufacture  3- foundry  4- glass industry  5- motor vehicle mechanic  6- wood worker  7- painter (already asked in Q19)  8- welder (already asked in Q18)  9- chemical industry  10- tannery  11- toolmaker or machine tool operator  12- miner or quarryman  13- insulation worker (already asked in Q20)  14- printing  15- meat workers  16- farmer  17- rubber industry  18- asbestos compounds production |
| COMPLETE THE APPROPRIATE SQs FOR THIS JOB AND MOVE TO THE GQ OF NEXT JOB | |

| *IRON AND STEEL PRODUCTION*  *(SQ1)* | Identification number: Job number: from year: to year: |
| --- | --- |
| Q1: Were you involved in the coke production? ………………………………... | *Y/N/DK*  *(if yes, please fill in the coke manufacture SQ instead of this SQ)* |
| Q2: Which of the following metals were produced in your factory? ……………….. | *Y/N/DK*  •pig iron  •mild steel  •stainless steel  •cadmium alloyed steel  if other alloyed steels, please specify: |
| Q3: Were you involved in ore processing? ……………………………….. | *Y/N/DK (if no go to Q4)*  if yes, in which part of the process?  *Y/N/DK Hours/Week*  •washing  •crushing, screening  •drying, sintering, calcinating, pelletizing  if other, please specify: |
| Q4: Were you working with a blast furnace? …………………………………… | *Y/N/DK (if no go to Q5)*  if yes, in which part of the process?  *Y/N/DK Hours/Week*  •charging of coke  •charging of iron  •recovery of molten iron and slag  if other, please specify: |
| Q5: Were you working with or near another kind of furnace? ………………… | *Y/N/DK (if no go to Q6)*  if yes, hours a week:  if yes, how was it powered?  •coke oven gas  •heavy oil  •natural gas  if other, please specify: |
| Q6: Were you working in one or more of the following sectors? ……………………. | *Y/N/DK Hours/Week*  •steel products manufacture  •electrode maintenance  •machine maintenance  •ovens maintenance  •production of by products from slag  •forging  if other, please specify: |
| Q7: If you were involved in the maintenance of furnaces, did you have to cut refractory blocks? …………………. | *Y/N/DK*  if yes, hours a week: |
| Q8: Did you harden metal parts? ……….. | *Y/N/DK Hours/Week*  •carburizing into furnaces  •cyaniding in a hot bath  •quenching in oil  if other, please specify: |
| Q9: Were you involved in the manufacture of plugging bolts? …………. | *Y/N/DK*  If yes, which of the following did you use?  *Y/N/DK Hours/Week*  •sand  •clay  •coal  •pitch  •synthetic resins  if other, please specify: |
|  | *END SQ1* |

| *WORKERS IN THE COKE PRODUCTION (SQ2)* | Identification number: Job number: from year: to year: |
| --- | --- |
| Q1: were you involved in work with coal? | *Y/N/DK*  if yes, hours a week:  if yes, was it:  *Y/N/DK Hours/Week*  •loading/unloading of coal  •crushing, mixing of coal  if other, please specify: |
| Q2: were you involved in work around a coke battery? ……………………………… | *Y/N/DK*  if yes, hours a week:  if yes, was it:  *Y/N/DK Hours/Week*  •heater  •top side worker (coal loader, lidsman, luterman...)  •bench side worker (doorman, pusher car ...)  •ash removal  •soot removal  •coke quenching  if other, please specify |
| Q3: were you involved in final work with coke (transportation, sizing, storage ...)? | *Y/N/DK*  if yes, hours a week: |
| Q4: Were you involved in the gas refining area? …...…………………………………... | *Y/N/DK*  if yes, hours a week:  if yes, were you involved in sulphur removing from coke gas?  *Y/N/DK*  if yes, hours a week: |
| Q5: Were you involved in the maintenance of ovens? ………………….. | *Y/N/DK*  if yes, hours a week:  if yes, did you have to cut refractory blocks?  *Y/N/DK*  if yes, hours a week: |
|  | *END SQ2* |

| *FOUNDRY WORKERS*  *(SQ3)* | Identification number: Job number: from year: to year: |
| --- | --- |
| Q1: What type of metals were melted in the foundry you worked in? ……………… | *Y/N/DK Hours/Week*  •cast iron  •mild steel  •stainless steel  •aluminium  •copper  •bronze  •lead  •cadmium  if other, please specify: |
| Q2: how were ovens powered in this foundry? …………………………………… | *Y/N/DK Hours/Week*  •coal  •coke  •electricity  if other, please specify: |
| Q3: In which part of the process were you involved? …………………………… | *Y/N/DK Hours/Week*  •sand preparation  •core/mould making  •metal melting  •pouring  •shaking out  •finishing  •maintenance  if other, please specify: |
| Q4: if you were involved in sand preparation, which materials were you mixing? …………………………………….. | *Y/N/DK Hours/Week*  •dry sand  •wet sand  •wood flour  •clay  •coal powder  •pitch  •mineral oils  •synthetic resins  if other, please specify: |
| Q5: Which core and mould sand binder systems were used in the foundry? ……. | *Y/N/DK Hours/Week*  •green sand  •sodium silicate sand (CO 2 process)  •sand binder with synthetic resins  if yes to synthetic resins, was it:  •phenol-formaldehyde resins (Ashland, pep-set, Croning process) *note for translation: use local equivalent*  •alkyd-isocyanates resins  if other, please specify: |
| Q6: Did you have to apply any coating on moulds? …………………………………… | *Y/N/DK*  if yes, hours a week:  if yes, which one? |
| Q7: if you were involved in shake out or finishing, which machine were you operating to clean castings? ……………. | *Y/N/DK Hours/Week*  •grinding wheels  •blow torch  •sand blasting  •steel shot blasting  if other, please specify: |
|  | *END SQ3* |

| *WORKERS IN A GLASS FACTORY (SQ4)* | Identification number: Job number: from year: to year: |
| --- | --- |
| Q1: In which part of the production were you personally involved? ………………… | *Y/N/DK Hours/Week*  •storage or mixing of raw materials  •melting  •blowing  •casting  •floating  •pressing  •drawing  •annealing  •finishing  •maintenance of kilns or furnaces  •maintenance of machines (for pressing ...)  if other, please specify: |
| Q2: If you were involved in storage or mixing of raw materials, which of the following? ………………………………….. | *Y/N/DK Hours/Week*  •dry sand  •wet sand  •soda ash  •potash  •fluorspar  •arsenic compounds  •chromium pigments  •cadmium pigments  if other, please specify:  if yes, how did you load, weigh, mix these raw materials?  *Y/N/DK Hours/Week*  •manually  •automatically without any ventilation  •automatically in a closed system |
| Q3: If you were involved in the maintenance of furnaces, did you have to cut refractory blocks? …………………. | *Y/N/DK*  if yes, hours a week: |
| Q4: Which kind of furnaces were used?.. | *Y/N/DK*  •electric  •gas  •fuel-oil  •coal  •coke |
|  | *END SQ4* |

| *WORKERS IN A GARAGE, CAR MECHANICS, REPAIRERS (SQ5)* | Identification number: Job number: from year: to year: |
| --- | --- |
| Q1: Which of the following tasks did you do? …………………………………………. | *Y/N/DK Hours/Week*  •diesel motor repairs  •gasoline motor repairs  •brake repairs:  if yes, did you use a high pressure  line to blow up dust?  •battery repairs  •body repairs  •tire repairs  if other, please specify: |
| Q2: Did you have to clean surfaces? … | *Y/N/DK*  if yes, was it:  *Y/N/DK Hours/Week*  •by burning  •with paint removers  •with acids  •with mineral oils  •with gasoline  if other, please specify:  If yes, which of the following coating were you removing ?  *Y/N/DK Hours/Week*  •tar or asphalt  •oils or greases  •putty  •paints  if other, please specify: |
| Q3: Did you polish or grind metal parts, with one or more of the following? ……... | *Y/N/DK Hours/Week*  •abrasive papers  if yes, was it:  -by hand  -with an electrical machine  •grinding wheels  •sandblasting  if yes, was it:  -with sand  -with steel shot  if other, please specify: |
| Q4: Did you use putty? …………………... | *Y/N/DK* if yes, was it:  *Y/N/DK Hours/Week*  •cellulose  •polyester  •bitumen *(blackson)note for translation: use the local name*  if other, please specify: |
| Q5: Did you use any machine tool? ……. | *Y/N/DK*  if yes, did you use cutting oils?  *Y/N/DK Hours/Week*  •straight oils  •soluble oils (soap oil)  •synthetic oils |
| Q6: Which of the following did you use to clean your hands or your material? …. | *Y/N/DK Hours/Week*  •gasoline  •hand cleaner  •mineral spirit  •paint thinner  •kerosene  if other, please specify: |
|  | *END SQ5* |

| *WOOD WORKERS /WOODWORK SHOPS (SQ6)* | Identification number: Job number: from year: to year: |
| --- | --- |
| Q1: Which of the following kind of wood working did you do? ……………………… | *Y/N/DK Hours/Week*  •sawmill  •wood treatment  •rough carpentry  •wood processing  •furniture  •floor laying  if other, please specify: |
| Q2: Which type of wood did you work with? ……………………………………….. | *Y/N/DK Hours/Week*  •hard wood  •soft wood  •plywood  •chipboard, pressed wood  if other, please specify: *(names of the main woods used)* |
| Q3: Which of the following machines did you use? …………………………………… | *Y/N/DK Hours/Week*  •steam-heated kiln (for wood treatment)  •saws (rip, cross-cut, band, panel)  •sanders, smoothers  if yes, was it with a dust bag?  •plywood or chipboard press  if other, please specify: |
| Q4: Did you treat wood with chemicals? . | *Y/N/DK*  if yes, hours a week:  if no, was the wood already treated? *Y/N/DK (if no go to Q5)* |
| Q4.1: which of the following did you use? | *Y/N/DK Hours/Week*  •creosotes  •tar and pitch  •copper, arsenic, chromium salts (CCA)  •chlorophenols  •fluor-chrome-arsenate phenol (FCAP)  if other, please specify: |
| Q4.2: How did you apply this wood treatment? ……………………………. | *Y/N/DK Hours/Week*  •spraying  •hot dipping  •cold dipping  •brushing, rolling  if other, please specify: |
| Q5: did you perform cleaning, pickling operations? ………………………………... | *Y/N/DK* if yes, hours a week:  if yes, which of the following did you use?  *Y/N/DK Hours/Week*  •acid solution  •paint remover  •mineral spirits  •by burning  if other, please specify: |
| Q6: Did you use varnishes, wood glazes, paints or wood stains? …………………… | *Y/N/DK* if yes, hours a week: |
| Q6.1 If yes, which of the following? …….. | *Y/N/DK Hours/Week*  •water-based paints  •solvent-based paints  •wood stains  •wood glazes  •alcohol based varnishes  other varnishes: |
| Q6.2: Which types of paints, varnishes, glazes did you use? ……………………… | *Y/N/DK Hours/Week*  •polyurethane  •urea-formaldehyde  •polyesters  •cellulosic  •glycerophtalic  if other, please specify: |
| Q6.3: How were the paints, varnishes, glazes applied? …………………………… | *Y/N/DK Hours/Week*  •spray  •brush / roller  if other, please specify: |
| Q7: Did you use adhesives? ...………….. | *Y/N/DK* if yes, hours a week:  if yes, which of the following?  *Y/N/DK Hours/Week*  •water based  •solvent based  •cellulose  •vinylic  •urea or phenol/formaldehyde  •polychloroprene  if other, please specify: |
| Q8: Did you use solvents or thinners? …. | *Y/N/DK* if yes, hours a week:  if yes, which of the following?  *Y/N/DK Hours/Week*  •mineral spirits  •trichloroethylene  •turpentine  •alcohol  if other, please specify: |
| Q9: Did you operate a laminating press? | *Y/N/DK* if yes, hours a week:  if yes, which adhesive did you use? |
|  | *END SQ6* |

| *PAINTERS/ WORKERS WHO PAINT (SQ7)* | Identification number: Job number: from year: to year: |
| --- | --- |
| Q1: Did you apply the paint on ………….. | *Y/N/DK Hours/Week*  •wood  •metal  •interior surfaces of building  •exterior surfaces of building  if other, please specify: |
| Q2: Did you clean up surfaces, before applying the paint, with one or more of the following? ……………………………... | *Y/N/DK Hours/Week*  •mineral spirits or paint thinners  •paint removers  •acid  •gasoline  •by burning  if other, please specify: |
| Q3: Which of the following coatings were you removing? ……………………………. | *Y/N/DK Hours/Week*  •tar or asphalt  •oils or greases  •putty  if yes which ones? |
| Q4: Did you polish or grind surfaces, before applying the paint, using? ………. | *Y/N/DK Hours/Week*  •grinding wheels  •sandblasting  if yes, was it:  -with sand  -with steel shot  •abrasive papers  if yes, was it:  -by hand  -with an electrical machine  if other, please specify: |
| Q5: Which of the following did you use? | *Y/N/DK Hours/Week*  •water based paints  •solvent based paints  •metal primers  •varnishes  •wood glazes  •wood stains  •wood preservatives  •asbestos filled paints  if other, please specify: |
| Q6: Which kind of paints did you use? … | *Y/N/DK Hours/Week*  •linseed oil  •acrylic  •vinyl  •alkyd (glycerophtalic)  •cellulose  •epoxy  •phenol/formaldehyde or urea/formaldehyde  •polyurethane  if other, please specify:  *note for translation: if possible, add usual names for some paints* |
| Q7: Which kind of solvents or thinners did you use for the paints? ………………. | *Y/N/DK Hours/Week*  •gasoline  •mineral spirit or paint thinner  •kerosene  if other, please specify: |
| Q8: Did you use rust-preventive paints or primers? …………………………………… | *Y/N/DK* if yes, with: *Y/N/DK Hours/Week*  •lead oxide  •lead chromate  •zinc chromate  if other, please specify: |
| Q9: How did you apply the paint? ………. | *Y/N/DK Hours/Week*  •spraying  •spraying in a spray booth  •dipping  •brushing / rolling  if other, please specify: |
| Q10: Where did you carry out painting? .. | *Y/N/DK Hours/Week*  •outdoors  •in a small room  •in a factory hall  if other, please specify: |
|  | *END SQ7* |

| *WORKERS PERFORMING WELDING, GAS CUTTING, BRAZING or SOLDERING (SQ8)* | Identification number: Job number: from year: to year: |
| --- | --- |
| Q1: Did you do soldering? ………………. | *Y/N/DK* If yes, was it with:  *Y/N/DK Hours/Week*  •soldering iron  •gas torch  •wave soldering  if other, please specify: |
| Q1.1: What kind of fluxes did you use? .. | *Y/N/DK Hours/Week*  •inorganic (with chlorides)  •rosin based  •organic, non rosin  if other, please specify: |
| Q2: Did you do gas welding or gas cutting or brazing? ………………………... | *Y/N/DK* if yes, which gas did you use:  *Y/N/DK Hours/Week*  •oxy-acetylene  •hydrogen  if other, please specify: |
| Q3: Did you do electric resistance welding? …………………………………… | *Y/N/DK*  if yes, hours a week: |
| Q4: Did you do electric arc welding? …... | *Y/N/DK*  if yes, was it:  *Y/N/DK Hours/Week*  •manual metal arc welding (MMA)  •arc welding with Tungsten electrode (TIG)  •semi-automatic arc welding (with gas shield / MIG or MAG)  •plasma arc welding  •submerged arc welding  if other, please specify: |
| Q5: What kind of metals did you assemble or cut? …………………………. | *Y/N/DK Hours/Week*  •cast iron  •mild steel  •stainless steel  •aluminium  •copper  •bronze  •lead  •cd plated  if other, please specify: |
| Q6: Did you weld metal pieces already coated with ………………………………... | *Y/N/DK Hours/Week*  •tar products  •grease or oils  •paints or primers  if other, please specify: |
| Q7: Did you clean metal parts, with one or more of the following? ……….………… | *Y/N/DK Hours/Week*  •mineral spirits or paint thinners  •paint removers  •gasoline  •acids  •by burning  if other, please specify: |
| Q8: Did you polish or grind metal parts, with one or more of the following: ……… | *Y/N/DK Hours/Week*  •abrasive papers  if yes, was it:  -by hand  -with an electrical machine  •grinding wheels  •sandblasting  if yes, was it:  -with sand  -with steel shot  if other, please specify: |
| Q9: Which of the following filler metal rods or electrodes did you use? ………… | *Y/N/DK Hours/Week*  •tin- lead alloy  •silver  •rutile  •basic  •cellulose  if other, please specify:  *note for translation: if possible, give examples of usual names, rod numbers etc.* |
| Q10: Where did you carry out welding? ... | *Y/N/DK Hours/Week*  •outdoors  •in small room (workshop)  •in factory hall  if other, please specify: |
| Q11: Did you perform X-Ray tests? …… | *Y/N/DK*  if yes, hours a week: |
|  | *END SQ8* |

| *WORKERS IN THE CHEMICAL INDUSTRY (SQ9)* | Identification number: Job number: from year: to year: |
| --- | --- |
| Q1: Were pesticides and / or fertilizers produced or packaged in your plant? ….. | *Y/N/DK*  *(if no go to Q2)*  if yes which ones?  *Y/N/DK Hours/Week*  •herbicides  •insecticides  •fungicides  •arsenic compounds  •fertilizers  If other, please specify:  *(do you remember any name: trade name, raw material..?)* |
| Q2: Were wood preservatives produced or packaged in your plant? ……………… | *Y/N/DK*  *(if no, go to Q3)*  if yes which ones?  *Y/N/DK*  •copper, arsenic, chromium salts (CCA)  •chlorophenols  •fluor-chrome-arsenate phenol (FCAP)  If other, please specify:  *(do you remember any name : trade name, raw materials, by products?)* |
| Q3: Were paints or varnishes produced or packaged in your plant? ……………… | *Y/N/DK*  *(if no go to Q4)* |
| Q3.1: If yes, which ones? …………..……. | *Y/N/DK*  •solvent-based paints  •water-based paints  •metal primers  •rust preventive paints  •varnishes  •wood glazes  •antifouling paints  •enamels  if other, please specify: |
| Q3.2: which of the following chemicals were produced? …………………………... | *Y/N/DK*  •linseed oil  •acrylic  •vinyl  •alkyd (glycerophtalics)  •cellulosic  •phenol resins (phenol/Formol or urea/Formol)  if other, please specify: |
| Q3.3: which of the following solvents were used? ……..………………………….. | *Y/N/DK*  •Turpentine  •Gasoline  •Mineral spirit or paint thinner  •Cellulosic thinner  If other, please specify the name: |
| Q3.4: were you exposed to organic dyes? | *Y/N/DK* If yes, hours a week:  if yes, do you remember any name? |
| Q3.5: were you exposed to dust from: …. | *Y/N/DK Hours/Week*  •pigments  if yes, was it:  -Chromium compounds (chromate...)  -arsenic compounds (Paris green...)  -cadmium compounds  -zinc compounds (zinc chromate)  -if other, please specify:  •fillers, extenders  if yes, was it:  -talc  -carbon black  -asbestos  if other dust, please specify: |
| Q3.6: do you remember any chemical or trade name used in your factory (raw material, by product ...) ? ………………... | specify: |
| Q4: Were polymers (for plastics, resins, synthetic rubbers or synthetic fibres) produced in your plant? ………………….. | *Y/N/DK (if no, go to Q5)* |
| Q4.1: If yes, which ones? ………………… | *Y/N/DK*  •polyethylene or polypropylene  •polyvinyl chloride  •polystyrene  •polyacrylate or polymethacrylate  •acrylonitrile- butadiene-styrene rubber (ABS)  •styrene-acrylonitrile rubber (SAN)  •polyacrylonitrile  •polyester  •polyamide  •urea or phenol /formaldehyde  if other, please specify: |
| Q4.2: which of the following did you use? | *Y/N/DK Hours/Week*  •plasticizers  •pigments  •organic dyes  •hardeners  •catalysts  •glass fibres  •fillers, extenders-  if yes, was it:  -carbon black  -talc  -asbestos  if other, please specify: |
| Q4.3: do you remember any name: trade names, raw materials, by-products? …… | specify: |
| Q5: Were synthetic fibres processed in your plant (by spinning ...) ? ….…………. | *Y/N/DK*  (if no, go to Q6)  if yes, which ones?  *Y/N/DK*  •polyamide (nylon ...)  •polyester (tergal ...)  •polyacrylate (Courtelle...)  *note for translation:use local trade names*  if other, please specify: |
| Q5.1 Do you remember any name: trade names, raw materials, by product? ….…. | specify: |
| Q6: Were adhesives produced or packaged in your plant? …………………. | *Y/N/DK (if no, go to Q7)* |
| Q6.1: If yes, which ones? ………………… | *Y/N/DK*  •solvent based  •water based  •latex adhesives  •hot melt adhesives  •cellulosic  •polyvinyl  •polyacrylate  •polychloroprene  •polyurethane  •urea or phenol/formaldehyde  if other, please specify: |
| Q6.2: Which of the following solvents or thinners were used? ……………………… | *Y/N/DK*  •mineral spirits  •ketones  •alcohol  if other, please specify: |
| Q6.3: Do you remember any name: trade name, raw material, by product? …..……. | specify: |
| Q7: Were any of the following used, produced or packaged in your plant? ….. | *Y/N/DK*  •cleaning agents  •disinfecting agents  •petroleum compounds  •dyes or pigments  •acids  if yes, do you remember any name : trade name, raw material, by product ... ? |
| Q8: Which of the following chemicals did you use or were you exposed to? ………. | *Y/N/DK Hours/Week*  •arsenic compounds  •chromium compounds  •cadmium compounds  •BCME  •acrylonitrile  •vinyl chloride  •ethylene oxide  •formaldehyde  •pigments  •organic dyes  •acid |
|  | *END SQ9* |

| *WORKERS IN A TANNERY (SQ10)* | Identification number: Job number: from year: to year: |
| --- | --- |
| Q1: In which parts of the process were you involved? …………………………… | *Y/N/DK Hours/Week*  •receipt of hides  •trimming  •soaking and washing  •fleshing  •unhairing/liming  •deliming  •pickling  •tanning  •retanning  •colouring  •coating  if other, please specify: |
| Q2: Did you use disinfecting agents? ….. | *Y/N/DK*  if yes, which of the following ?  *Y/N/DK Hours/Week*  •DDT  •chlorophenols  •formaldehyde (gas or liquid)  •arsenic compounds  if other, please specify: |
| Q2.1: Did you weigh, mix, prepare these disinfecting baths yourself? ……………… | *Y/N/DK* |
| Q3: Did you use tanning agents? ………. | *Y/N/DK*  if yes, which of the following?  *Y/N/DK Hours/Week*  •vegetable tanning  •synthetic tanning  •formaldehyde tanning  •chrome tanning  if yes, was it a:  -one bath chrome tanning (chromium sulphate)  -two bath chrome tanning (bichromate) |
| Q3.1: Did you weigh, mix, prepare these tanning baths yourself? …………………... | *Y/N/DK*  if yes, hours a week: |
| Q4: Did you spray leather in the finishing area? ………………………………..……… | *Y/N/DK*  if yes, hours a week:  if yes, which of the following did you spray?  *Y/N/DK Hours/Week*  •acrylic resins  •polyurethane resins  •coloured resins  •mineral oils  •fungicides  if other, please specify: |
| Q5: Did you use pigments or dyes? ……. | *Y/N/DK*  if yes, which of the following?  *Y/N/DK Hours/Week*  •black pigments  •coloured pigments  •cadmium pigments  •lead pigments  •organic dyes  if other, please specify: |
| Q5.1: Did you weigh, mix, prepare these pigments or dyes yourself? ………………. | *Y/N/DK*  if yes, hours a week: |
|  | *END SQ10* |

| *TOOL MAKERS and MACHINISTS (SQ11)* | Identification number: Job number: from year: to year: |
| --- | --- |
| Q1: Which of the following machine tools did you use? ……………………….……… | *Y/N/DK Hours/Week*  •lathe  •milling machine  •planing machine  •boring machine  •drilling machine  •grinding machine  •sawing machine  •sharpening machine  if other, please specify: |
| Q2: Which of the following materials were you machining? ………………………….... | *Y/N/DK Hours/Week*  •copper  •bronze  •brass  •lead  •aluminium  •cast iron  •mild steel  •stainless steel  •galvanized steel  •cadmium plated steel  •plastic  if other, please specify: |
| Q3: Did you make or repair tools or pieces made of the following materials? | *Y/N/DK Hours/Week*  •tungsten carbide  •high carbon steel  •high speed steel  •chromium oxide  if other, please specify: |
| Q4: Did you clean up surfaces with one or more of the following? ………………… | *Y/N/DK Hours/Week*  •mineral spirits or paint thinners  •paint removers  •acid  •by burning  if other, please specify: |
| Q4.1: If yes to one or more cleaning products, how did you perform this cleaning? …………………………………... | *Y/N/DK Hours/Week*  •with a brush, a roller ...  •by dipping in a cold bath  •by dipping in a hot bath  if other, please specify: |
| Q4.2: If yes to one or more cleaning products, which of the following coatings were you removing? …..………………… | *Y/N/DK Hours/Week*  •tar or asphalt  •oils or greases  •putty  •paints  if other, please specify: |
| Q5: Did you polish or grind surfaces using? ……………………………………... | *Y/N/DK Hours/Week*  •abrasive papers  if yes, was it:  -by hand  -with an electrical machine  •grinding wheels  •sandblasting  if yes, was it:  -with sand  -with steel shot  if other, please specify: |
| Q6: Did you use cutting oils? …………… | *Y/N/DK*  if yes, which of the following?  *Y/N/DK Hours/Week*  •straight oils  •soluble oils (soap oil ?)  •synthetic oils |
| Q7: Did you harden metal parts? ……… | *Y/N/DK*  if yes, which of the following?  *Y/N/DK Hours/Week*  •carburizing into furnaces  •cyaniding in a hot bath  •quenching  if yes, in:  -water  -oil  if other, please specify: |
|  | *END SQ11* |

| *MINERS and QUARRYMEN (SQ12)* | Identification number: Job number: from year: to year: |
| --- | --- |
| Q1: Did you work in ………………………. | *Y/N/DK Hours/Week*  •an opencast mine  •a quarry  •underground |
| Q1.1: If it was underground, was radioactivity measured in your mine? …... | *Y/N/DK* |
| Q2: What material was mined, quarried? | *Y/N/DK*  •iron  •copper  •lead  •nickel  •coal  •asbestos  •clay  •marble  •granite  •stones  •sand  if other, please specify  *(note for translation: depending on the local mining, add other usual materials in this list)* |
| Q3: Which of the following tasks did you perform? …………………………………… | *Y/N/DK Hours/Week*  •blasting (shot-fire)  •underground roofing  -if yes,  -with wood  -with metal  •sampling  •extraction  •mineral crushing or milling  if other, please specify: |
| Q4: Did you operate any of the following machines? ………………………………... | *Y/N/DK Hours/Week*  •cutting machine  •drilling machine  •pneumatic drills  •boring machines  •loading machines (bulldozer...)  •crushing machine  •jig  •floatation machine  •shuttle car  •conveyor belt  if other, please specify: |
| Q4.1: If you were operating drilling or cutting machines, was this work done dry? ………………………………………… | *Y/N/DK*  or with water injection?  *Y/N/DK* |
| Q5: If you performed blasting, which kind of explosives did you use? ………………. | •Ammonium-nitrate/fuel oil (anfo)? *Y/N/DK*  if other, please specify: |
| Q6: How were pneumatic equipment of the mine powered? ……………………….. | *Y/N/DK*  •by an engine on the floor  •by a diesel motor underground  if other, please specify: |
|  | *END SQ13* |

| *WORKING WITH INSULATION MATERIALS or FIBER PANELS (SQ13)* | Identification number: Job number: from year: to year: |
| --- | --- |
| Q1: Where were the insulation materials or fiber panels installed? ………………… | *Y/N/DK Hours/Week*  •around pipes  •ovens, boilers  •buildings  •electrical equipment  if other, please specify: |
| Q2: Which of the following materials were you in contact with? ………………...…….. | *Y/N/DK Hours/Week*  •fibreglass  •mineral wool  •polystyrene  •polyurethane foam  •asbestos  •ceramic fibres  •urea/formaldehyde foam  •polyurethane foam  if other, please specify:  *note for translation: give examples of trade names or usual names when possible* |
| Q3: If you install yourself insulating materials, how did you do it? …………… | *Y/N/DK Hours/Week*  •by injection of foam  •splattering  •blown up of powder  •rigid panels  •pipe sheathing  if other, please specify: |
| Q4: Were you installing or removing these materials in an enclosed space (under a roof ...) without any natural or mechanic ventilation? …………………….. | *Y/N/DK*  if yes, hours a week: |
| Q5: Did you have to cut or make holes in these materials? ………………………….. | *Y/N/DK*  if yes, hours a week:  if yes, was it: *Y/N/DK Hours/Week*  -by hand  -with electric powered machines |
| Q6: Did you have to work with cement, concrete? ………………………………….. | *Y/N/DK*  If yes, for which tasks? *Y/N/DK Hours/Week*  •making holes in concrete  •covering insulation with cement  •using a concrete mixer  if other, please specify: |
|  | *END SQ14* |

| *PRINTING (SQ14)* | Identification number: Job number: from year: to year: |
| --- | --- |
| Q1: Which printing process was used in your workshop? …………………………… | *Y/N/DK Hours/Week*  •letterpress  •lithography /offset  •gravure  •silk screen process  if other, please specify: |
| Q2: In which part(s) of the process were you involved? ……………………………… | *Y/N/DK Hours/Week*  •manual or mechanical composition  -if yes, were you involved in lead melting?  •development of images  •photo engraving process  •electroplating  •printing  •bookbinding  if other, please specify: |
| Q3: Which task(s) did you perform? ….… | *Y/N/DK Hours/Week*  •sand blasting (of metal blocks or plates ...)  •steel shots blasting  •coating with a photosensitive layer (of paper sheets, metal plates, screens ...)  •varnishing (of cylinders, metal plates ...)  •acid etching (of a plate, a block, a cylinder...)  •cleaning (of cylinders, screens, photo engraving plates ...)  •electroplating  if other, please specify: |
| Q4: Which of the following did you handle or were you exposed to? |  |
| Q4.1: Inks: …………………………………. | *Y/N/DK*  if yes, were you exposed  *Y/N/DK Hours/Week*  •from your working environment  •from a self use  was it:  •viscous (or fat) ink  •thin inks  •black inks  •coloured inks  do you remember some names of dyes or pigments?  •carbon black  •chromate  •cadmium compounds  •if other, please specify:  did you have to mix by yourself pigments or dyes? *Y/N/DK*  if yes, hours a week: |
| Q4.2: Cleaning solvents or thinners: ……. | *Y/N/DK*  if yes, hours a week:  if yes, were you exposed  *Y/N/DK Hours/Week*  •from your working environment  •from self use  was it :  •chlorinated solvents  •mineral spirits  •alcohols  •gasoline  •kerosene  if other, please specify: |
| Q4.3: Chemicals for photosensitive layer: | *Y/N/DK*  if yes, hours a week:  (for metal plates, screens or insulating paper)    if yes, were you exposed :  *Y/N/DK Hours/Week*  •from your working environment  •from a self use of metal plates  •from screens or insulating paper already coated  •while coating by yourself metal plates,  •from screens or insulating paper  if other, please specify:  *Y/N/DK*  was it :  •gum  •dichromate  •photo sensitive polymers  if other, please specify: |
| Q4.4: Acids: …..…………………………… | *Y/N/DK*  if yes, hours a week:  if yes, were you exposed  *Y/N/DK Hours/Week*  •from your working environment  •from a self use  to etch metal plates or cylinders  for electroplating chromium plating  nickel plating  Copper plating  *Y/N/DK*  Was it:  •chlorhydric acic  •chromic acid  •sulfuric acid  •acetic acid  •nitric acid  if other; please specify: |
|  | *END SQ15* |

| *MEAT WORKERS/ SLAUGHTERERS (SQ15)* | Identification number: Job number: from year: to year: |
| --- | --- |
| Q1: Please indicate the animals being slaughtered or processed, and the number in your work area ……………….. | *Y/N/DK How many a week*  •pigs  •cattle  •sheep and lambs  •goats  •chickens, turkeys  •horses  if other, please specify: |
| Q2: Did you perform any of the following tasks on whole carcass? ………………… | *Y/N/DK % of job*  •receiving/ holding of animal  •stunning / killing  •bleeding  •head / shank removal  •skinning / dehairing / defeathering  •evisceration  •splitting / cutting carcass  •washing / scalding  •chilling  •deboning  •disposal of carcasses  •overwrapping and packaging  if other, please specify: |
| Q3: Did you come in close contact with any of the following? ……………………… | *Y/N/DK % of job*  •feathers/ hair  •blood  •faeces, urine  •viscera |
| Q4: Did you perform any of the following meat processing operations? ……….…… | *Y/N/DK % of job*  •cutting  •mincing  •curing  •smoking  •cold smoke process  •hot smoke process  •packing  if other, please specify: |
| Q5: Did you wrap meat in plastic film? … | *Y/N/DK* if yes, % of job: |
| Q6: Was the plastic film cut with a hot wire? ……………………………………….. | *Y/N/DK* |
| Q7: Did you use preservatives on the meat? ………………………………………. | *Y/N/DK* if yes, what type: |
| Q8: Did you use rat or mice poisons? …. | *Y/N/DK* if yes, what type: |
| Q9: Have you ever developed any warts on your hands? …………………………… | *Y/N/DK*  if yes, when did you develop these?  if yes, for how long did they last? |
|  | *END SQ15* |

| *FARMERS, GARDENERS (SQ16)* | | | Identification number: Job number: from year: to year: | | |
| --- | --- | --- | --- | --- | --- |
| Q1: How would you classify your farm/workplace? ………………………….. | | | *Y/N/DK* Size of farm (Hectares)  •arable farm  •animal husbandry  •mixed arable/animals  •market gardening  •horticulture/flower growing  •vineyards  •orchards  •gardening / park keeping  •forestry  if other, please specify: | | |
| Q2: What livestock was raised where you worked? ………………………………. | | | *Y/N/DK* Average number of animals  •none *(If none jump to Q7)*  •milk cows  •cattle for meat  •pigs  •sheeps, goats  •chickens  •other poultry  if other, please specify: | | |
| Q3: Were you involved in feeding the animals? …………………………………… | | | *Y/N/DK* | | |
| Q3.1: If yes, what did you feed them? ….. | | | *Type of animal Feed*  •  •  • | | |
| Q4: Were the animals slaughtered on the farm? ………………………………….……. | | | *Y/N/DK*  if yes, did you personally slaughter the animals *Y/N/DK*  if yes, did you personally butcher the animals *Y/N/DK* | | |
| Q5: Did any infectious epidemics occur among the animals at the farm during your employment there? ………………… | | | *Y/N/DK* | | |
| Q5.1: If yes, which epidemics? ………….. | | | *Epidemic When (year)*  •  •  • | | |
| Q6: Were you involved in disinfecting the animals? …………………………………… | | | *Y/N/DK* | | |
| Q6.1: If yes, what treatment did you usually apply? ……...……………………… | | | *type of animal treatment times per year*  •  •  • | | |
| Q7: For crop growing, what crops were you involved in growing, handling or spraying? ………………………………….. | | | *Y/N/DK*  •none  •wheat  •corn  •rice  •barley  •other cereals (please specify)  •vineyard  •olive tree  •sugar beet  •soyabean, sunflower  •strawberries  •apple or pear trees  •citrus fruit/trees  •other trees  •mushrooms  •tobacco  •vegetables  •flowers  if other, please specify: | | |
| Q8: Did you work in greenhouses with the following? …………………………………... | | | *Y/N/DK*  •greenhouse vegetables  •greenhouse flowers  •mushrooms  if other, please specify: | | |
| Q9: Were any herbicides, insecticides or fungicides (pesticides) used on the farm/garden / forest? …………………….. | | *Y/N/DK*  if yes, complete the following questions | | | |
|  | | | | |  |
| Q9.1: Herbicides | *Herbicide 1 Herbicide 2 Herbicide 3*  A-In which year were the treatments first applied?  B-In which year were the treatments last applied?  C-How many years in total?  D-Did you personally apply the treatments?  E-How many days per year on average?  F-How many hours (av) did you spend on the pesticide treatments, on those days?  G-Did you prepare the mixture?  H-Can you recall the names of the products?  I-Can you recall the seeds or crops or animal feeds which were being treated?  J-Can you recall the pests, weeds or infections which were being treated?  K-How were the treatments applied, (i.e. backpack or hand sprayer, tractor mounted spray, aerial mounted spray)?  L-Did you use any protective equipment when applying the pesticides (overalls/ working clothes/ handkerchief over mouth/ filter mask/ cabin on tractor)? | | | | |
| Q9.1: insecticides | *Arsenic compounds Other Insecticides 1 Other Insecticides 2*  A-In which year were the treatments first applied?  B-In which year were the treatments last applied?  C-How many years in total?  D-Did you personally usually apply or assist in the treatments?  E-How many days per year on average?  F-How many hours (av) did you spend on the pesticide treatments, on those days?  G-Did you prepare the mixture?  H-Can you recall the names of the products?  I-Can you recall the seeds, crops or animal feeds which were being treated?  J-Can you recall the pests, weeds or infections which were being treated?  K-How were the treatments applied, (i.e. backpack or hand sprayer, tractor mounted spray, aerial mounted spray)?  L-Did you use any protective equipment when applying the pesticides (overalls/ working clothes/ handkerchief over mouth/ filter mask/ cabin on tractor)? | | | | |
| Q9.2: fungicides | *Fungicides 1 Fungicides 2 Fungicides 3*  A-In which year were the treatments first applied?  B-In which year were the treatments last applied?  C-How many years in total?  D-Did you personally usually apply or assist in the treatments?  E-How many days per year on average?  F-How many hours (av) did you spend on the pesticide treatments, on those days?  G-Did you prepare the mixture?  H-Can you recall the names of the products?  I-Can you recall the seeds, crops or animal feeds which were being treated?  J-Can you recall the pests, weeds or infections which were being treated?  K-How were the treatments applied, (i.e. backpack or hand sprayer, tractor mounted spray, aerial mounted spray)?  L-Did you use any protective equipment when applying the pesticides (overalls/ working clothes/ handkerchief over mouth/ filter mask/ cabin on tractor)? | | | | |
| Q10: Did you apply fumigants? ……………………………………………………………... | | | | *Y/N/DK* | |
| Q11: Did you clean the equipment, or the tools used for the pesticide treatments? …. | | | | *Y/N/DK* | |
| Q11.1: If yes, did you use petrol or other solvents? ………………………………….…... | | | | *Y/N/DK* | |
| Q12: Did you apply any wood preservatives to fences yourself? ……………………….. | | | | *Y/N/DK* | |
| Q12.1: If yes, what type? ………………… | | | describe: | | |
|  | | | *END SQ16* | | |

| *WORKERS IN THE RUBBER INDUSTRY (SQ17)* | Identification number: Job number: from year: to year: |
| --- | --- |
| Q1: Which of the following types of rubber was produced? …………………… | *Y/N/DK*  *•*natural rubber  *•*synthetic, acrylonitrile styrene butadiene (ABS)  *•*synthetic, styrene acrylonitrile (AN rubber)  *•*synthetic, styrene butadiene (SB rubber)  if other, please specify: |
| Q2: Did you or any of your neighbours work with raw material (storage, weighing, mixing, compounding, loading...)? ………………………………… | *Y(himself)/Y(neighbours)/N/DK* |
| Q2.1: If yes, which of the following compounds did you or your neighbour handle? ..…………………………………… | *Y/N/DK*  *•*rubber  *•*clay  *•*carbon black  *•*pigments  *•*dyes  *•*oils  *•*coal tar  if other additions, please specify: |
| Q3: Did you or any of your neighbours operate rolling mills, calendars, press or extruders? …………………………………. | *Y(himself)/Y(neighbours)/N/DK* |
| Q4: Were you or any of your neighbours involved in the curing process? …………. | *Y(himself)/Y(neighbours)/N/DK* |
| Q5: Did you or any of your neighbours use antitack products? …………………… | *Y(himself)/Y(neighbours)/N/DK* |
| Q5.1: If yes, was it……..………………….. | *Y/N/DK*  *•*by powdering  *•*by dipping  *•*by spraying  was it:  *•*talk  *•*clay  *•*soapstone  if other, please specify: |
| Q6: Did you or any of your neighbours use petroleum oils? ………………………. | *Y(himself)/Y(neighbours)/N/DK* |
| Q6.1: If yes, was it…………………………. | *Y/N/DK*  *•*by dipping  *•*by spraying |
| Q7: Did you or any of your neighbours mix rubber with a solvent (to make a paste, a glue…)? ……………….…………. | *Y(himself)/Y(neighbours)/N/DK* |
| Q7.1: If yes, was it…………………………. | *Y/N/DK*  *•*benzene  *•*gasoline  *•*mineral spirit  if other solvent, please specify: |
| Q8: In which of the following sectors/departments of activity/production were you involved? … | *Y/N/DK Hours/Week*  *•*production line  *•*control room  *•*laboratory  *•*maintenance of buildings  *•*maintenance of machines  *•*cleaning of workshops  *•*packaging  if other, please specify: |
|  | *END SQ17* |

| *ASBESTOS COMPOUNDS PRODUCTION (SQ18)* | Identification number: Job number: from year: to year: |
| --- | --- |
| Q1: Were you or a neighbour in the same workshop, involved in debagging asbestos fibers? ………………………….. | *Y(himself)/Y(neighbours)/N/DK* |
| Q2: Were you or a neighbour in the same workshop, involved in milling asbestos? .. | *Y(himself)/Y(neighbours)/N/DK* |
| Q3: Were you or a neighbour in the same workshop, involved in textile operations on asbestos fibres (carding, spinning, weaving…)? ……………………………….. | *Y(himself)/Y(neighbours)/N/DK* |
| **Q4**: Were you or a neighbour in the same workshop, involved in mixing asbestos? .. | *Y(himself)/Y(neighbours)/N/DK* |
| Q4.1: If yes, was it with…………………... | *Y(himself)/Y(neighbours)/N/DK*  *•*sand  *•*cement  *•*resin/plastic (UF/PF…)  if other, please specify: |
| Q5: Were you or a neighbour in the same workshop, involved in removing asbestos mixed parts from a mould? …... | *Y(himself)/Y(neighbours)/N/DK* |
| Q5.1: If yes, which kind of machines or tools were used …………………………… | *Y(himself)/Y(neighbours)/N/DK*  *•*hand tools  *•*blower  if other, please specify: |
| Q6: Were you or a neighbour in the same workshop, involved in heating, curing asbestos mixed compounds? …………… | *Y(himself)/Y(neighbours)/N/DK* |
| Q7: Were you or a neighbour in the same workshop, involved in machining dry asbestos products? ……………..………... | *Y(himself)/Y(neighbours)/N/DK* |
| Q7.1: if yes, was it ………………...…...… | *Y(himself)/Y(neighbours)/N/DK*  *•*by turning  *•*by cutting  *•*by drilling  *•*by grinding  if other, please specify:  did you do the machining using:  *•*hand tools  *•*electric tools  if other, please specify: |
| Q8: Were you or a neighbour in the same workshop, involved in finishing asbestos products, applying any of the following compounds: …………………………….…. | *Y(himself)/Y(neighbours)/N/DK*  *•*paint  *•*coal tar or pitch  if other, please specify:  If yes to one of the above, was this applied with  *•*with a brush  *•*with a sprayer  if other, please specify: |
| Q9: Were you or a neighbour in the same workshop, involved in cleaning the working area, machines, moulds or other materials? …………………………………. | *Y(himself)/Y(neighbours)/N/DK* |
| Q9.1: If yes, was it with …………...……… | *Y(himself)/Y(neighbours)/N/DK*  *•*a brush  *•*a blower  if other, please specify: |
| Q10: Were you working in the waste disposal part of the factory? …………….. | *Y/N/DK* |
| Q10.1: if yes, was it ………...…………….. | *Y/N/DK*  *•*dry fibres waste  *•*wet waste  *•*lump waste  If other, please specify: |
|  | *END SQ18* |
